# One night with Venus, a lifetime with mercury: Analyses of heavy metals in Franz Schubert’s hair are consistent with syphilis treatment

**DOI:** 10.1007/s00508-025-02524-8

**Published:** 2025-04-29

**Authors:** Lisa Fischer, Stephan Hann, Christina Amory, Walther Parson, Farkas Pintér, Christian Reiter

**Affiliations:** 1 Institute of Analytical Chemistry, BOKU University, Muthgasse 18, 1190 Vienna, Austria; 2 Institute of Legal Medicine, Medical University of Innsbruck, Muellerstraße 44, 6020 Innsbruck, Austria; 3 Institute of Conservation, University of Applied Arts Vienna, Salzgries 14, 1010 Vienna, Austria; 4 Experts Office for Forensic Medicine, Diepoldplatz 10, 1170 Vienna, Austria; 5 Medical University of Vienna, Vienna, Austria

**Keywords:** Biomonitoring, Forensic analysis, Time-resolved hair analyses, Heavy metal load, LA-ICP-SFMS

## Abstract

Samples from three strands of Franz Schubert’s hair, which are very likely to be authentic due to their well-documented origin and the chain of custody that is still valid today, were analyzed in terms of molecular biology and heavy metal content. The two hair samples presumably taken from the deceased’s head showed matching molecular biological parameters, while the sample taken 35 years after his burial did not contain any usable DNA. Heavy metal analyses along the hair samples using LA-ICP-MS (laser ablation inductively coupled plasma mass spectrometry) with a temporal resolution of 4–5 h revealed significantly increased mercury and lead concentrations in all samples with a decreasing tendency towards the time of death. Taking the different storage conditions into account, the patterns of metal concentrations in the samples match well, suggesting that all three samples came from the same individual. The results of our research support the suggestion, often made in the literature, that Franz Schubert underwent cutaneous treatment with mercury for syphilitic illness many months before his death.

**Supplementary Information:**

The online version of this article (10.1007/s00508-025-02524-8) contains supplementary material, which is available to authorized users.

## Introduction

When Franz Schubert, an Austrian composer born on 31 January 1797, fell ill in early November 1828, his state of health was probably not considered as life threatening by those around him. Written documentation about the symptoms of disease let his relatives and friends assume that he suffered from an infectious illness. Nevertheless, many of these conducted several sickbed visits, whereas some, like his friend Franz von Schober, may have held back out of prudence [1]. Dr. Josef Ritter von Vering on behalf of Schubert’s physician Dr. Ernst Rinna von Sarenbach [2] diagnosed blood dyscrasia on 13 November 1828 [3–5] after conducting a phlebotomy. Dr. Johann Baptist Wisgrill was consulted several days later (18 November) [1]; however, he could not prevent the dramatic deterioration of Schubert’s health within a few days. Franz Schubert died of typhoid (nebulous) fever on 19 November 1828, in his brother Ferdinand’s apartment in the newly built house at the address Auf der Neuen Wieden N° 694, today Kettenbrückengasse 6, 1040 Vienna [6]. There have been numerous articles on Schubert’s medical history and speculation about the cause of his death, with some questioning whether he actually died of “nervous fever” (a synonym for typhoid fever/typhus abdominalis at the time), as a consequence of a latent/recurrent secondary stage of syphilis or from mercury poisoning [7–9]. With human remains such as hair (and teeth or bones), reliable insights into a death event could be obtained by applying highly sensitive state-of-the-art analytical techniques [10]. Contrary to the assumption that Schubert’s hair had fallen out during sickness and respective treatment, reports describe that his “rather large, round and coarse head was surrounded by brown, luxuriantly sprouting curly hair” and “full brown, naturally curling hair” [11]. After Ludwig van Beethoven passed away in 1827, many strands of his hair were removed from his skull by numerous admirers [12]. Schubert’s corpse though has been protected from such interventions and only a few locks of hair were removed from his head at the time of his death. Also, during the first exhumation on 13 October 1863, several tufts of his hair, which were already dissociated from the head, were saved [13]. More information regarding the provenance is given in the supplementary section of this article.

Via mediation by Prof. Mag. Helena Dearing we received a few strands of these valuable relicts for the analyses of spatially distributed heavy metal concentrations. Our results may contribute to the clarification of the cause of death of Franz Schubert.

## Material and methods

### Provenance of Schubert’s hair

In the following section, provenance and proof of authenticity of the samples is described briefly. More information can be found in the supplementary section.

Hair samples of curls which are currently in the private possession of Raimund Hofbauer (Kritzendorf, Lower Austria) and preserved in a sand-colored envelope have also been provided for analysis. An inscription, written in German current handwriting (style approximately from 1850) declared the authenticity: *“For Mrs. Mitzi Schubert, retired teacher, curl from Fr. Schubert. By kindness—*a synonym for delivered by messenger at the time” [translated].

**Fig. 1:**
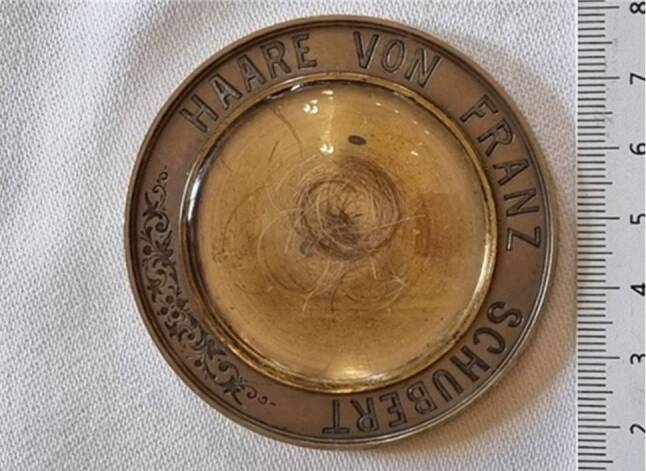
Photograph of the medallion with Franz Schubert’s hair. Light-colored bronze, diameter 64 mm, circular engraving

A few single hairs were removed with sterile and pre-annealed tweezers, transferred into small paper envelopes and labelled accordingly: *Schubert A 1.W, Schubert A 2.W, Schubert A 3.W, Schubert B* and *Schubert B 1.W.* A lock of Franz Schubert’s hair, which originates from the collection of Ignaz Weinmann [14], is on loan from the Vienna City Library and currently exhibited at the Schubert Memorial Site in the castle Atzenbrugg, Lower Austria. A picture of the medallion is depicted (see Fig. 1) which is labelled as follows:

“Medallion with Franz Schubert’s hair. Light-coloured bronze, diameter 64 mm, circular engraving ‘Hair of Franz Schubert’. In the centre a depression (diameter 44 mm) containing hair remnants mixed with wood particles. Domed cover glass above.” (WBR, MS, ZPM 338, Bibliotheca Schubertiana Ignaz Weinmann)

Two single hairs of the loose strands of approximately 3 and 4 cm in length were removed from the medallion individually using sterile and annealed splinter tweezers and subsequently transferred in paper envelopes labelled as follows: *Schubert C 1—tox* and *Schubert C 2—DNA.*

### Mitochondrial (mt) DNA analysis

Three single hairs were provided for molecular genetic analyses applying mtDNA analysis. Hair C was submitted in two segments with approximately 1.5 cm (without root) and 1 cm (with root) in length. The root portions of hair A (approx. 2.3 cm) and hair B (approx. 7 cm) were excised. The shaft of hair B was cut into 1 cm long sections prior to DNA extraction and root and shaft segments were investigated independently. In the first step, the surface of the hair shafts (not roots) was cleaned with aqua bidest. and then subjected to a mild lysis. The protocol consists of incubation (2 h, 56 °C) in 900 µL of a lysis buffer (10 mM Tris pH 8.0, 100 mM NaCl, 1 mM CaCl_2_, 2% SDS, 36 µl proteinase K) under agitation to remove external contamination. Full lysis of the purified hair shafts and uncleaned roots was performed overnight in 900 µL of the lysis buffer (see above), 10 µL proteinase K and 10 µL 1 M DTT. On the following day, 10 µL proteinase K and 10 µL 1 M DTT were additionally added and the samples were incubated until no hair structures were visible anymore. The DNA extraction was performed following a modified protocol developed by Dabney et. al which is outlined in [15]. In our study, 2 mL binding buffer and 80 µL 3 M sodium acetate were used. The mtDNA analysis was performed according to the PEC protocol detailed in [16]. Haplogrouping was performed using SAM2 via EMPOP following the procedure outlined in [17]. Quantification of nuclear DNA and mtDNA was performed using the SDquant system [18].

### Hair analysis by LA-ICP-SFMS

Laser ablation inductively coupled plasma sector field mass spectrometry (LA-ICP-SFMS) has provided a valuable tool for obtaining spatial information of samples with a high lateral resolution, high sensitivity and multielement capability. Single hairs were analyzed from the root of the hair to the tip using a NWR213™ Nd:YAG 213 nm laser ablation system (ESI Elemental Scientific Inc., Omaha, NE, USA) coupled to an ICP-SFMS (Element 2, Thermo Fisher Scientific, Bremen, Germany). The ablated material was transported out of the ablation cell in a continuous flow of helium (550 mL min^−1^). The carrier gas was mixed with argon (1.2–1.25 L min^−1^) via a Y-connection of the transport line and the analyte-gas mixture was directed into the ICP. Laser scanning was performed with a spot size of 50 µm in a first scan and 20 µm in a second scan in the same line with a scan speed of 50 and 10 µm s^−1^. The applied laser energy (fluence) was 27.8–29.1 J cm^−2^. The ICP-SFMS was operated in low mass resolution mode (*m/∆m* = 300). Masses monitored were: ^34^S, ^75^As, ^111^Cd, ^121^Sb, ^195^Pt, ^200^Hg, ^202^Hg and ^208^Pb. Data acquisition was performed in E‑scan mode (50% mass window, 60 samples per peak, 10 ms sampling time), resulting in a cycle time per pass of 3.524 s (50 µm s^−1^ scan speed) and 1.924 s (10 µm s^−1^ scan speed). For the best possible focusing of the laser beam onto the sample surface, individual sections of approx. 10 mm were analyzed with an overlap of 200 µm between the individual sections. Each measurement cycle started with the acquisition of a blank. The average of the obtained blank intensities was subtracted from the subsequent analyte signal. In this study, no absolute quantification of the investigated elements was performed. Instead, the signal intensities obtained for the investigated samples were assessed via comparison to those obtained for hair samples from healthy individuals. Sulfur was used as internal standard for normalization of the metal signals as its concentration has been reported to be relatively constant in hair [19]. Prior to analysis, all hair samples were precleaned by soaking in 1:10 diluted NovaClean™ AFX (pure11 GmbH, Grünwald, Germany) general purpose cleaner for 1 h, rinsing with ultrapure water followed by acetone in three cycles and drying in a laboratory oven at 75 ± 5 °C overnight. The hair samples were adhered onto glass slides using double-sided tape “Pritt non-permanent”. Starting points and directions of the analysis were indicated on the glass slide. Thus, growth directions and the arrangement of the time coordinates could be subsequently confirmed using SEM analyses.

### Scanning electron microscopy (SEM)

Scanning electron microscopy (SEM) is a powerful method to investigate the microstructure and topography of both inorganic and organic matter. To obtain detailed information about the state of condition of the strands of hair, the samples investigated by LA-ICP-SFMS were subsequently analysed with a JEOL JSM-IT200 scanning electron microscope (JEOL (Germany) GmbH, Freising, Germany), under high vacuum. In order to preserve the samples in their original appearance, the hairs were not coated by carbon prior to the investigation. To avoid charging and damage caused by the electron beam on the surface of the nonconductive hairs and simultaneously achieve images with a reasonable resolution and quality, a very low acceleration voltage (i.e., 1 kV) and a slow speed of scanning process (50 s) were used.

## Results

### Mitochondrial (mt) DNA analysis

**Fig. 2:**
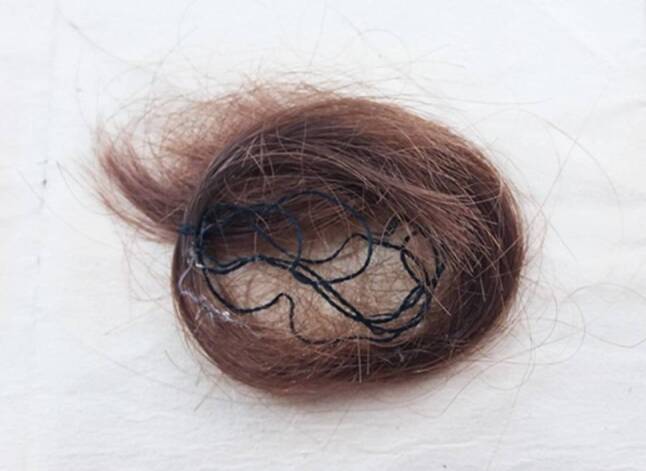
Curls of Franz Schubert (Private possession of Raimund Hofbauer, Kritzendorf, Lower Austria)

Molecular genetic analyses were carried out on three individual hair samples. Their authenticity is based on independently documented sampling processes. The mtDNA and DNA concentrations were very low as expected for such old hair fragments, except for the root of hair A (Table 1). While the shaft segments were precleaned, the roots were not cleaned as the cleaning procedure would have removed genuine (mt)DNA from the root. Therefore, root portions are more likely to show extraneous contamination. Indeed, the root portion of hair A resulted in higher sequence read depths than the shaft portions and also the mitotype differed. This mitotype was also present in our local contamination exclusion database which contains mtDNA sequences of staff, technical personnel, students and visitors. These results suggest that the higher values obtained for the root portion of hair A could represent contamination. The remaining hair segments showed higher values for the 69 bp target than for the 143 bp target which is in line with expectations for severely degraded DNA in such old specimens [16]. Hair segments which yielded > 69 bp were subjected to mtDNA sequencing. Hair segments A‑S and B‑S yielded mtDNA concentrations of approx. 100 mitogenomes µL^−1^ and resulted in mitotypes which cannot be excluded as deriving from the same individual and lineage (Table 1). Hair C‑S did not yield sequencing results above the required reads. The identical mitotype 263G 315.1 C 750G 15784C 16519C (range 1–841, 8211–8476, 15784–16569) which was shared by hair A‑S and hair B‑S belonged to haplogroup H65. This mitotype was observed 9 times in a database of 75,838 mitogenomes (mitoTree; Nicole Huber, Institute of Legal Medicine, Medical University of Innsbruck, Innsbruck, Austria, personal communication), which equals 0.0145% (augmented counting method; x + 2 / *n* + 2, where *x* is the number of observations and *n* is the database size). Applying a 99.5% confidence interval (BetaInv) results in an upper limit of 0.0232% or 1:4300 for the probability that a randomly selected individual matches this mitotype. Despite the lack of maternally related reference samples from a (living) relative of Franz Schubert, we can assume that the two curls in Raimund Hofbauer’s possession come from one and the same person (Fig. 2).

**Table 1:** Results of mtDNA and DNA analysis and mitotypes

Sample	Mt69 [mtG µL^−1^]	Mt143 [mtG µL^−1^]	nDNA [pg µL^−1^]	Mitotype (relative to rCRS)
Hair A‑R	205.6	117.3	5.85	73G 150T 152C 195C 215G 263G 295T 311T 315.1 C 315.2 C 319C 489C 513A 573.1 C 750G 16069T 16126C 16145A 16231C 16240R 16261T
Hair A‑S	109.7	1.3	n.r.	263G 315.1 C 750G (8745G) (8860G) 15784C 16519C
Hair B‑R	42.2	n.r.	0.46	n. s.
Hair B‑S	103.3	0.8	0.07	263G 315.1 C 750G (15784C) 16519C
Hair C‑R	n.r.	n.r.	0.10	n. s.
Hair C‑S	n.r.	n.r.	0.27	n.r.
Ex0	n.r.	n.r.	0.27	n.r.
Lib-NC	–	–	–	n.r.

Sequence interpretation ranges:

Hair A‑R: 1–853 8240–8374 15852–16569

Hair A‑S: 1–879 8161–8476 (8578–8887) 15747–16569

Hair B‑S: 1–841 8211–8480 (15784) 15785–16569

Parentheses indicate low read depth

Variants and ranges in brackets indicate sequence read depth below 20 reads

*R* root, *S* shaft, *mtG* mitogenome equivalent, *mt69* mtDNA fragment 69 bp, *mt143* mtDNA fragment 143 bp, *nDNA* nuclear DNA 70 bp, *rCRS* revised Cambridge Reference Sequence, *n.r.* no result, *n.* *s.* not sequenced, *Ex0* extraction blank, *Lib-NC* library negative control

### Hair analysis by LA-ICP-SFMS

**Fig. 3:**
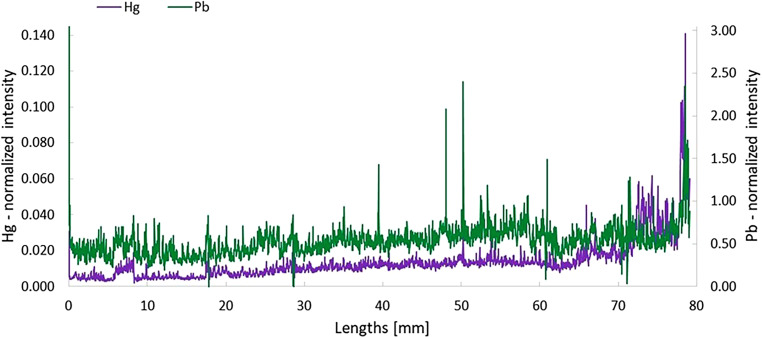
Variations of relative concentrations (^34^S normalized intensities) of Hg and Pb in a hair sample of Franz Schubert (Schubert A 3.W). The hair sample was ablated from close to the root (0 mm) to the end of the hair (surface scan), reflecting exposure of toxic metals during approximately the last 8 months of his life

**Fig. 4:**
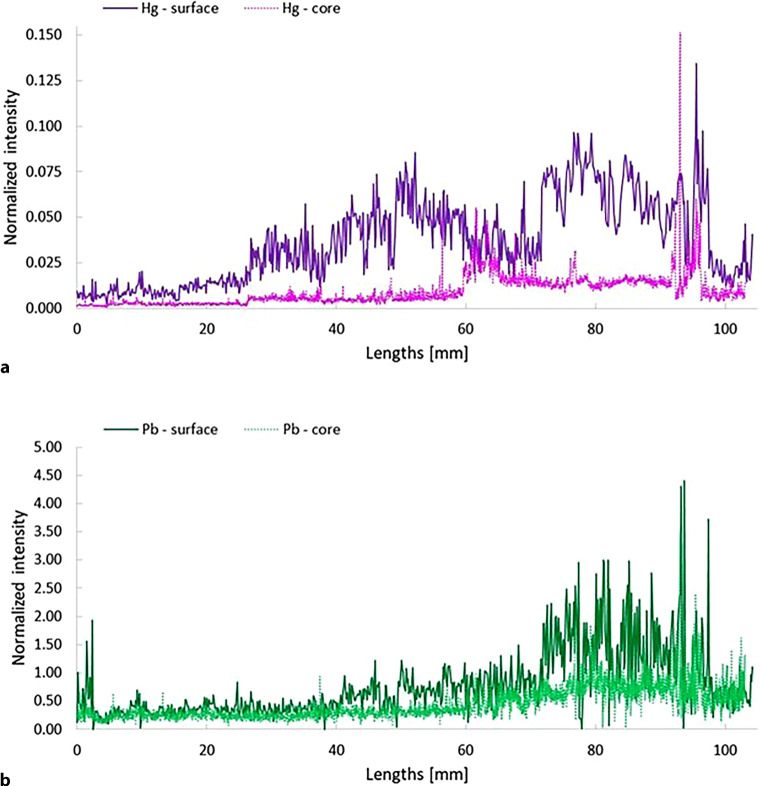
Variations of relative concentrations (^34^S normalized intensities) of Hg (**a**) and Pb (**b**) in the surface (cuticle) and the core of a hair sample of Franz Schubert (Schubert B). The hair sample was ablated from close to the root (0 mm) to the end of the hair, whereas the second scan was performed with an offset of the z‑axis (10 µm) to laser scan the inner part of the hair

The LA-ICP-SFMS is the method of choice for sensitive and selective measurement of the distribution of trace elements in hair samples [20]. The hair sample is placed in a closed ablation chamber which is continuously passed through by a carrier gas. The laser beam with defined energy and size ablates, i.e., vaporizes, a certain amount of material, which is directly transported to the ICP-MS where the ablated material is atomized, ionized and detected after mass spectrometric separation in real time. To monitor longitudinal variations of relative elemental concentrations in the individual hairs, the obtained transient analyte signals were converted to hair length and lifetime. Assuming an average hair growth rate of 10 mm per month, a time resolution of approximately 9.8 h (50 µm s^−1^ scan speed) and 1.4 h (10 µm s^−1^ scan speed) has been obtained. Analyte/sulfur ratios of controls (unexposed healthy individuals with natural hair color) were calculated and compared to those obtained from the hair samples of Franz Schubert. In view of the very limited amount of hair samples available, it was not possible to determine the absolute concentrations of mercury or lead by acid digestion. Figure S1 (supplementary section) shows the relative intensities of mercury (Hg) and lead (Pb) in a single hair of a male nonsmoking individual, obtained by ablating the sample from the root to the end of the hair at the surface (cuticle). Average ratios of 0.0025 ± 0.0004 (^202^Hg/^34^S) and 0.0008 ± 0.0004 (^208^Pb/^34^S) were calculated. Variations of these metals longitudinally in a hair sample of Franz Schubert (*Schubert A 3.W*) are depicted in Fig. 3. A significant decrease in concentrations, in particular 8–5 months before his death, clearly reflect exposure of toxic metals. Elevated concentrations of mercury and lead up to a factor of 50 and 2000, respectively (signal spikes were considered as outliers and thus, not included), were detected during growth of all hair samples analysed. Figure 4 shows relative intensities of mercury and lead in the cuticle (surface scan) and the core of a hair sample *(Schubert B)* from close to the root to the tip of the hair. Decreased concentrations in the core relative to the cuticle could be determined for these elements, whereby the greatest difference in relative concentrations was observed for mercury.

### Scanning electron microscopy (SEM)

**Fig. 5:**
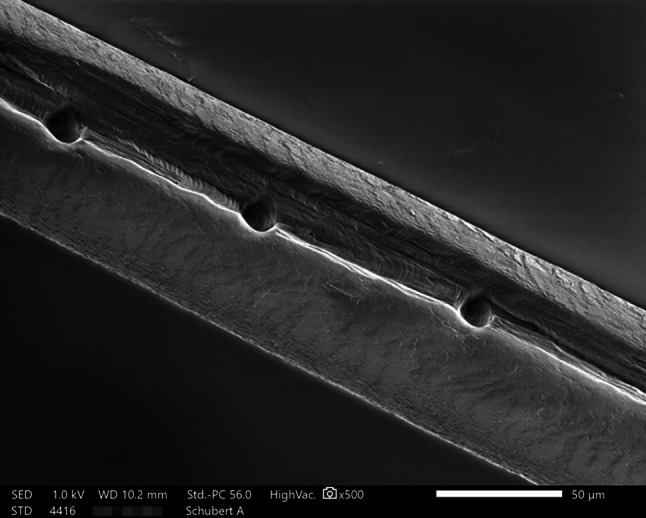
SEM image of a section of Schubert B hair sample after LA-ICP-SFMS analyses, showing the 20 µm wide longitudinal groove in the cortex and the 10 µm wide punch-outs in the core of this hair of a hair. *LA-ICP-SFMS* laser ablation inductively coupled plasma sector field mass spectrometry

The SEM analysis showed that even at low magnifications it could be clearly observed that the laser had milled a 20 µm wide longitudinal groove into the cortex of all hairs, exposing the medulla. In addition, individual measurements taken at 80 μm intervals revealed regular punch-outs of 10 μm in the exposed hair pith, providing data at time intervals of approximately 5–6 h (see Fig. 5).

Detailed examinations of the hairs from the two Schubert/Hofbauer locks showed a thickness of 46–95 μm and a well-preserved scale structure of the hair surfaces. There were no impurities. Hairs from the medallion (Weinmann/Vienna City Library), on the other hand, showed a severe loss of scale structure of the surface and a fluctuating hair thickness between 45 and 75 μm. Extensive microbial contamination and spongy, fine-pored matrix loosening was found both on the surface and in the medullary substance.

Finally, the observations by SEM enabled determination of the arrangement of the cuticle, consequently, the direction of hair growth could clearly be determined in all samples. This information was of outmost importance, because it helped to verify the results of the LA-ICP-SFMS analysis (i.e., the direction of the spot measurements related to the direction of the growth of the hairs).

## Discussion

In addition to environmental and industrial exposure, significant uptake and accumulation of heavy metals in the body occur through inhalation of plant pyrolysis products. This is the reason why their concentrations in biological samples including hair and nails are significantly higher in smokers compared to nonsmokers [21]. Hair samples of our healthy, nonsmoking controls, age 30–40 years, showed average relative concentrations of 0.0025 for ^202^Hg/^34^S and 0.001 for ^208^Pb/^34^S, while concentrations determined in hair from a medium to heavy smoker were in the range of 0.002–0.003 for ^202^Hg/^34^S and 0.005–0.01 for ^208^Pb/^34^S. Presumably, Franz Schubert occasionally smoked a pipe [5]; however, relative concentrations of mercury and lead detected in his hairs, which were up to 50 and 2000 times higher, respectively, compared to controls, cannot be solely explained by tobacco consumption.

Mercury and compounds containing mercury were medicinally applied to treat syphilis infections from the sixteenth century until the early twentieth century [22]. In Schubert’s time, syphilis treatment with mercurial ointment liniments (metallic mercury dispersed in grease) were commonly applied and also practiced in the General Hospital of Vienna [23, 24]. Applying this treatment in closed, unventilated and warm spaces, the elemental mercury vapor, which occurs even at (higher) room temperatures due to the volatility of the element, was inhaled, absorbed by the lungs and diffused in the bloodstream. Symptoms of intoxication such as increased salivation indicated effectiveness of the treatment in those times. Endogenous mercury enters the hair follicle and binds to *alpha-*keratin and associated proteins due to the high affinity of mercury to the thiol groups of the amino acid cysteine via linear coordination of divalent mercury [25, 26]. Thus, hair can be used as a significant biomarker for heavy metal exposure. Elevated mercury levels in the hair samples of Franz Schubert were evenly distributed over the entire length of approximately 7–10 cm, reflecting approximately the last 10 months of his life. There is no indication regarding mercury-based medication within the last few months preceding his death and medicines taken 2 weeks before his death were not specified on the prescription [5]; however, in cases of typhoid fever (formerly known as “nervous fever”), doctors would not have prescribed medication containing mercury. Furthermore, a significant increase of the relative mercury concentration would have been observed in the hair samples over the last 2 weeks, corresponding to 0–5 mm of lengths. Elemental/metallic mercury affects all body tissues via the blood circulation, consequently, it bioaccumulates in the central nervous system, the kidneys and, due to the liposolubility, it crosses the blood-brain barrier and accumulates in the brain, particularly if it is inhaled. The biological half-life of mercury is approximately 30–60 days, whereas in the brain, it might be 20 years or even more as oxidized elemental mercury strongly binds to the Se and SH groups of biological ligands [27, 28]. Constant release of bioaccumulated mercury from body tissues into the bloodstream and incorporation into the hair matrix over many years could explain the elevated relative concentrations in the hair of Franz Schubert.

In the early nineteenth century, lead acetate was used as a local disinfectant and astringent to flush out secretions from the urethra [29]. The absorption of lead through inflamed mucous membranes or open skin injuries were accepted as a side effect. In the early stages of syphilis and gonorrhea (these diseases were not separated until the nineteenth century) lead acetate solutions were used, which could have led to bioaccumulation in the body. Almost all absorbed and circulating lead binds to erythrocytes and is thus transported to soft body tissues. Due to its chemical similarity to calcium, lead is incorporated into the mineral bone matrix. The degree of bone-incorporated lead is age-dependent and trabecular and cortical bone of adults can store up to 95% of retained lead. The half-life of lead in bone is estimated to be 20–30 years, whereas the half-life in blood is only about 35 days [30]. Lead turn-over in bone is naturally slow and can be increased in certain life situations, hence, toxicological effects can occur long after lead exposure due to bone turnover and remobilization of lead, which may explain the elevated relative concentrations of lead in Franz Schubert’s hair.

Up to the time of his death, no signs of serious vital deficits were reported, apart from headaches and a certain weakness. This could be reconciled with allusions in the correspondence of his friends in the period around 1823, where he seems to have been “treated at the General Hospital” [5] and his scalp hair had to be shaved “because of a skin eruption” [1]. The recurrent headaches and some weakness in the last years of his life may also be related to post-therapeutic exposure to lead [31]. Even at sub-toxic levels in the blood, mercury and lead have serious effects on hematopoiesis and the immune system [32, 33].

As shown in Fig. 4a, b and the relative concentrations of mercury and lead were higher in the surface (cuticle) than in the core of the hair samples and vary similarly over time. Lower metal concentrations in the core compared to the cuticle are in accordance with previous findings [34]; however, the reason for this peculiarity remains unclear. Variations of mercury in the outer and inner area of sample B (see Fig. 4a, in particular in the section from 60–70 mm) do not show the same variation. This decrease in signal intensity could be explained by diagenetic changes, microbial degradation and the associated loss of heavy metals in parts of the outer hair layers of the Schubert B sample with a corresponding decrease in concentrations until the time of death.

## Conclusion

The continuous but on average 50-fold increase of the relative mercury concentration and the up to 2000-fold increase of the relative lead concentration in Schubert’s hair in the last 10 months before his death indicate a massive heavy metal exposition years before with subsequent long-term excretion. Accordingly, Schubert’s fatal “nervous fever” and its course must therefore also be assessed in the context of pre-existing heavy metal exposure and the resulting hematological and immunological consequences. As a general conclusion, the significantly increased mercury and lead concentrations in Schubert’s scalp hair can be most likely explained by a previous syphilis treatment.

## Supplementary Information

Supplementary

Figure S1: Relative concentrations (^34^S normalized intensities) of heavy metals in a single hair (cuticula) of a male non-smoking individual. The hair sample was ablated from the root (at 0 mm) to the end of the hair.
