# Supplementary material for: One night with Venus, a lifetime with mercury: Analyses of heavy metals in Franz Schubert’s hair are consistent with syphilis treatment
Source: Wien Klin Wochenschr. 2025 Apr 29;137(13-14):438–45. doi: 10.1007/s00508-025-02524-8 (PMC12241130; doi:10.1007/s00508-025-02524-8)
Supplement: Supplementary file 1 — Supplementary [file 508_2025_2524_MOESM1_ESM.docx]

### Supplementary

#### Supplementary information regarding provenance and proof of authenticity of Schubert’s hair samples

The assumption, that Franz Schubert suffered from hair loss due to sickness and medication treatment can be disproved by numerous contemporary illustrations pictured by the artists Wilhelm August Rieder, Moritz Ludwig von Schwind, Leopold Kupelwieser, Ferdinand Georg Waldmüller, Josef Eduard Teltscher and Anton Depauly throughout his life until shortly before his death.

Austrian sculptor Josef Alois Dialer, who manufactured the portrait bust for the grave of Franz Schubert at the town cemetery Währing, and physician Louis Kainzlberger (Kaindlsberger; the spelling of the name conflicts in different statements) are said to have removed curls of Schubert at his death [^[[1]](#endnote-1)^, ^[[2]](#endnote-2)^]. The curls which were kept by Dialer, came into possession of Schubert’s grandniece Marie Schubert [1] and are currently in private possession of the Hofbauer family (Kritzendorf, Lower Austria) as a result of recurring inheritance. The relic, owned by Dr. Kainzlberger, came into possession of Franz von Hartmann (Linz, Upper Austria) and his children and was sold to Greece by the Antiquarian V. A. HECK, Vienna in 1928 [^[[3]](#endnote-3)^].

During the first exhumation of Schubert’s corpse on October 13^th^ 1863, 35 years after his death [^[[4]](#endnote-4)^], his brother, Andreas Schubert received several tufts of Franz Schubert´s hair. Later, he gave them away (mostly with notarisation) [2]. Amongst others, music collector Ignaz Weinmann was in possession of one of these locks, before he consigned it to the City of Vienna [^[[5]](#endnote-5)^]. As stated in the main article, this valuable relict is now exhibited at the Schubert Memorial Site in the castle Atzenbrugg (Lower Austria).

*Material and sample collection*

**Hair from Marie Schubert/Hofbauer family**

An envelope, sand-coloured, 15.5 x 10.1 cm, with the inscription in German Current script *"For Mrs Mitzi Schubert, retired teacher, curl from Fr. Schubert. By kindness (=Through messenger)"* contained a folded letter, sand coloured, 20.2 x 12.7 cm with the inscription *"Franz Schubert 1828"* and *"16/Sch/2"*. The letter contained two curls of hair lying on top of each other (see Figure 2 in the main article) inofficially labelled by us as curl *A* and *B*.

Curl *A* consists of individual hairs, whereas hairs from Curl *B* were tied together with a black string approximately halfway along the length. Few white down feathers were found close to the knot of the string. The curls exactly correspond to those shown in Deutsch [1]. Curl *A* contains approximately twice as many hairs as curl *B* with estimated total lengths of 10 and 9 cm, respectively. The colour of the hair is a light chestnut with a shade of red (golden brown). Inspection with a microscope did not show any impurities apart from down feather deposits. Individual hairs were of different thickness and did not show mechanical damage. In curl *A*, three hairs were found with well-preserved roots. The curls were handed over to C. Reiter on February 21^st^ 2023, in presence of Raimund Hofbauer, his wife, son Nikolaus and Helena Dearing in Kritzendorf (Lower Austria) for sample collection for further analysis. Samples *Schubert A 1.W* (ca. 8 cm*), Schubert A 2.W* (ca. 3 cm), *Schubert A 3.W* (ca. 10 cm), *Schubert B* (ca. 10 cm, without root) and *Schubert B 1.W* (ca. 9 cm, crushed root) were taken (see main article). The remaining curls were returned in their original packaging to Raimund Hofbauer on April 17^th^ 2023.

**Hair from the Weinmann collection/Vienna City Library**

A document and invoice stored at the Vienna City Library provided with the sale of the medallion with hair from Franz Schubert (see Figure 1 in the main article) confirm it’s provenance. It is read as follows:

*“Medallion with hair by Franz Schubert probably from the possession of Andreas Schubert, to whom they were given on the occasion of Franz Schubert's exhumation on 13 October 1863.*

*Purchased on 19 November. 1927, from Josef Rosenstingl, antique dealer, Vienna, 1st, Spiegelgasse 25 (Allegedly acquired shortly before from a jeweller's family in Vienna, 1st, Kohlmarkt).*

*Replacement glass. The flat original glass, easily movable in the metal frame, broke during transport; replacement glass (purchased from: Anton Köhler und Sohn, Vienna, 15., Pelzgasse 12) inserted in the same month (November 1927).*

*Cleaning. Cleaned of light mould coating in August 1936 by Mr Skalitzky, preparator at the Museum of Ethnology, Vienna, 1st, Neue Hofburg, - i.e.: first treated with fresh carbolic acid, then brushed over with paraffin, the glass cleaned with alcohol and sealed with yellow sticky wax.*

*(Through the kind mediation of Mrs Emilie Anders, Vienna, 4th, Floragasse 5.)*

*Photographic reproduction, scale 1:1, made ca. 1929 by ?*

Invoice: *“Antiquariat LÖCKER & WÖGENSTEIN, dated 25.8.93, addressed to Dr. Steblin named: Ign.Weinmann, collection - Schubert, according to offer 12000-, cheque” (*[rechnung-loecker.jpg (395×565) (michaelorenz.at)](https://michaelorenz.at/schubertlocke/rechnung-loecker.jpg).

Following mediation and official approval by the former artistic head of the Schubert Memorial Site, Prof. Mag. Helena Dearing, the medallion was opened on March 14^th^ 2023 by art conservator Amelie Bezard in presence of Stefan Engl (head of the department of music at the Vienna City Library), Christian Reiter and Helena Dearing.

The domed cover glass could be lifted off without damage. Individual hairs were found inside, some partly stuck together, some separated with an average length of 3 - 4 cm. The hair colour varied from light brunette to chestnut brown. Inspection with a strong magnifying glass did not show any impurities such as wood particles or insect remains as originally stated in a further description, which is available in the Vienna City Library. Hair with hair roots could not be found. Samples Schubert C 1 – tox, Schubert C 2 - DNA and Schubert C 3 (spare, not analysed) were taken (see main article). These samples were handed over to Christian Reiter for chemical and molecular-biological analysis. In presence of all people involved in sampling, the remaining hairs were resealed with the cover glass and adhesive by the art conservator.

1. Deutsch O.E. (1913) Franz Schubert. Sein Leben in Bildern. München und Leipzig, Georg Müller. [↑](#endnote-ref-1)
2. Hilmar E., Jestremski M. (1997) Schubert-Lexikon „Haare“ Akad. Druck- u. Verlegeanstalt Graz. [↑](#endnote-ref-2)
3. Antiquariat V. A. Heck (1928?) Katalog (Nr. 46): Autographen-Sammlung Dr. F.C. Witte, Rostock nebst einigen Beiträgen aus anderem Besitz - Wien: Autographen-Handlung V. A. Heck. DOI: https://doi.org/10.11588/diglit.68124#0002. [↑](#endnote-ref-3)
4. Freiherr von Helfert J-A (1863) Aktenmäßige Darstellung der Ausgrabung und Wiederbeisetzung der irdischen Reste von Beethoven und Schubert. Carl Gerold's Sohn Verlag. [↑](#endnote-ref-4)
5. Lorenz M. (2010) "The Johann Strauss Odyssey" - A Few Necessary Notes. Web link: [Michael Lorenz: Replik auf Walburga Litschauers Beitrag "Perspektiven der Schubert-Forschung in Österreich" (michaelorenz.at)](https://michaelorenz.at/mattl-wurm/english.htm). [↑](#endnote-ref-5)
