# Supplementary figures and images for: One night with Venus, a lifetime with mercury: Analyses of heavy metals in Franz Schubert’s hair are consistent with syphilis treatment
Source: Wien Klin Wochenschr. 2025 Apr 29;137(13-14):438–45. doi: 10.1007/s00508-025-02524-8 (PMC12241130; doi:10.1007/s00508-025-02524-8)

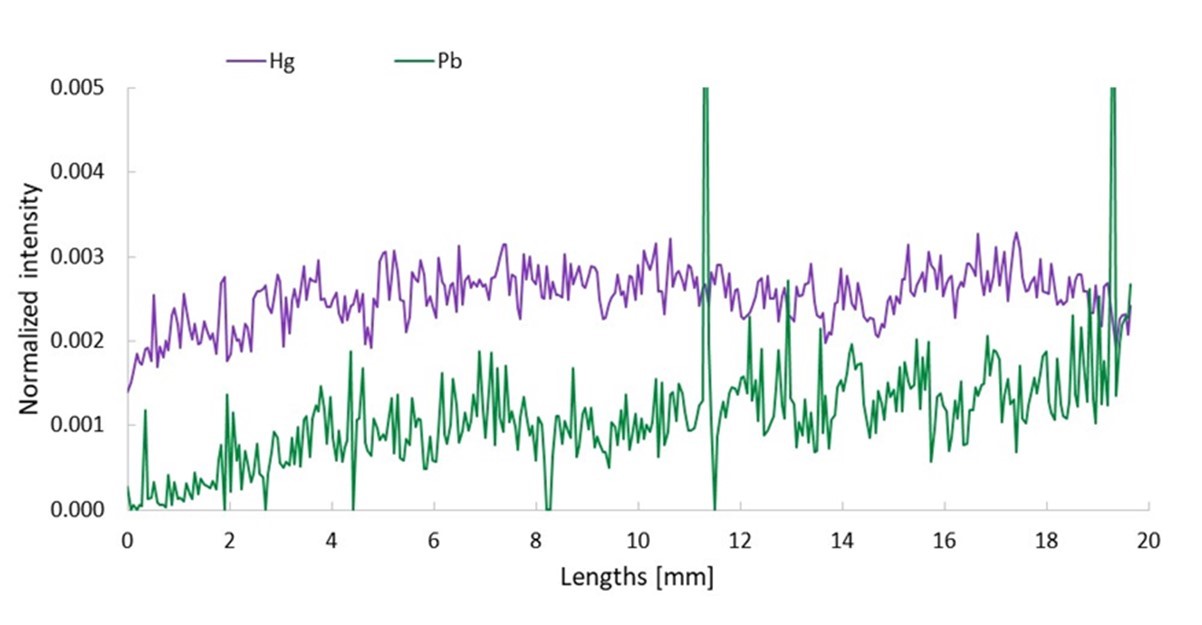

Supplement: Supplementary file 2 — Figure S1: Relative concentrations (34S normalized intensities) of heavy metals in a single hair (cuticula) of a male non-smoking individual. The hair sample was ablated from the root (at 0 mm) to the end of the hair. [file 508_2025_2524_MOESM2_ESM.jpg]
